# Detection of impending perfusion deficits by intraoperative computed tomography (iCT) in aneurysm surgery of the anterior circulation

**DOI:** 10.1007/s00701-021-05022-8

**Published:** 2021-10-13

**Authors:** Jun Thorsteinsdottir, Torleif Sandner, Annamaria Biczok, Robert Forbrig, Sebastian Siller, Patricia Bernasconi, Andrea Szelényi, Thomas Liebig, Jörg-Christian Tonn, Christian Schichor

**Affiliations:** 1 Department of Neurosurgery, Ludwig-Maximilians-University, Campus Grosshadern, Marchioninistr. 15, 81377 Munich, Germany; 2 Department of Neuroradiology, Ludwig-Maximilians-University, Campus Grosshadern, Marchioninistr. 15, 81377 Munich, Germany; 3 Department of Anesthesiology, Ludwig-Maximilians-University, Campus Grosshadern, Marchioninistr. 15, 81377 Munich, Germany

**Keywords:** Intraoperative computed tomography, Aneurysm, Clipping, CT perfusion, CT angiography

## Abstract

**Background:**

The aim of our study was to evaluate the additional benefit of intraoperative computed tomography (iCT), intraoperative computed tomography angiography (iCTA), and intraoperative computed tomography perfusion (iCTP) in the intraoperative detection of impending ischemia to established methods (indocyanine green videoangiography (ICGVA), microDoppler, intraoperative neuromonitoring (IONM)) for initiating timely therapeutic measures.

**Methods:**

Patients with primary aneurysms of the anterior circulation between October 2016 and December 2019 were included. Data of iCT modalities compared to other techniques (ICGVA, microDoppler, IONM) was recorded with emphasis on resulting operative conclusions leading to inspection of clip position, repositioning, or immediate initiation of conservative treatment strategies. Additional variables analyzed included patient demographics, aneurysm-specific characteristics, and clinical outcome.

**Results:**

Of 194 consecutive patients, 93 patients with 100 aneurysms received iCT imaging. While IONM and ICGVA were normal, an altered vessel patency in iCTA was detected in 5 (5.4%) and a mismatch in iCTP in 7 patients (7.5%). Repositioning was considered appropriate in 2 patients (2.2%), where immediate improvement in iCTP could be documented. In a further 5 cases (5.4%), intensified conservative therapy was immediately initiated treating the reduced CBP as clip repositioning was not considered causal. In terms of clinical outcome at last FU, mRS0 was achieved in 85 (91.4%) and mRS1-2 in 7 (7.5%) and remained mRS4 in one patient with SAH (1.1%).

**Conclusions:**

Especially iCTP can reveal signs of impending ischemia in selected cases and enable the surgeon to promptly initiate therapeutic measures such as clip repositioning or intraoperative onset of maximum conservative treatment, while established tools might fail to detect those intraoperative pathologic changes.

## Introduction

Aneurysm clipping is a routine procedure with low complication rates in interdisciplinary neurovascular centers [12, 21, 36]. Complete aneurysm occlusion, preservation of the parent, and the efferent vessels are the primary goals during surgery. However, intraprocedural complications like aneurysm rupture or ischemia might impact on patient outcome [22]. The rate of treatment-related ischemia during aneurysm clipping was reported to be approximately 11% in the prospective ISUIA study [36]. Technical efforts in detecting imminent ischemia due to relative clip stenosis, parent vessel occlusion, or thromboembolic events have been made. Current available intraoperative imaging modalities—like indocyanine green videoangiography (ICGVA) [8, 10, 28, 29] or intraoperative DSA (iDSA) [1, 6, 9]—turned out to be helpful in this context. ICGVA shows aneurysm remnants, parent vessel reconstruction, and patency of small perforating arteries in close vicinity of the clipped aneurysm within the microscopical field [8, 10, 28, 29]. As ICGVA is limited to the visual field, possible entrapment and affection of distant vessels, e.g., by improper clip placement, may be missed [32]. iDSA provides information about the local anatomy of the clip construct and anatomical preservation of dependent vessels with a high resolution [1, 6, 9]. However, this method is invasive and requires special logistic effort. In addition to those intraoperative imaging modalities, intraoperative monitoring (IONM) of motor- and somatosensory evoked potentials (MEPs/SEPs) predicts postoperative deficits related to the monitored neuronal pathways with a high specificity [33, 34]. However, it does not provide information about the location of the affected vessel or the source of critical perfusion deficit. In our former series, IONM did not necessarily improve overall outcome in all clipping procedures, but it proved to be helpful in selected cases [13].

Recently, we demonstrated in a small cohort of patients that intraoperative computed tomography (iCT) including angiography (iCTA) and perfusion (iCTP) was feasible without disrupting the intraoperative workflow. Modern iCT systems nowadays offer similar image resolution to conventional CT scanners in radiology departments and include artifact suppression software and the possibility of performing iCTA/iCTP [11]. While iCTA defines the parent vessel anatomy post-clipping, iCTP visualizes critical distant perfusion impairment in case of clip stenosis by quantifying cerebral blood perfusion (CBP) within a very short acquisition time [30–32].

The aim of this study was to evaluate whether multimodal iCT during the clipping procedure has a relevant impact on the detection of perfusion deficits and on intraoperative decision-making compared to established methods like ICGVA, microDoppler, and IONM.

## Methods

### Patients

Data on 93 patients who underwent microsurgical aneurysm clipping in an operating room equipped with a “double room CT scanner” [23] were prospectively collected between October 2016 and December 2019. Patients harboring aneurysms of the posterior circulation were excluded as the detection of infratentorial mismatches, especially of small volume perfusion deficits in the brainstem, by iCTP is limited [5]. All patients received intraoperative ICGVA, microDoppler examination, and pre- and postoperative digital subtraction angiography including 3D reconstruction (DSA). Upon availability intraoperative monitoring including MEP and SEP was performed.

Patient characteristics included age, sex, rupture status, multiple aneurysms, Fisher grade (in case of a subarachnoid hemorrhage), and neurological status. Aneurysm-specific characteristics comprehended side, location, and size. Procedure-related variables such as temporary clipping, microvascular Doppler ultrasonography, ICGVA, and critical changes in IONM variables were documented. Adjustments in operative management due to suspected perfusion deficits were monitored: visual inspection of vascular segment and clip position, clip repositioning, or the initiation of medical intervention. iCT data, including unenhanced brain CT, CTA, and CTP, were meticulously analyzed for relevant surgical information and need for intervention. In addition, pre-/postoperative angiography, IONM, ICGVA, and subsequent imaging data (CT and/or MRI) were retrospectively reassessed during hospitalization. Clinical outcome was assessed postoperatively using the modified Rankin scale (mRS) at discharge and subsequent follow-up (FU) visits. The study was approved by the institutional review board (No.18–281). Informed consent was obtained from all patients.

### Clinical protocol

All patients underwent preoperative assessment by 3D-DSA. Preoperatively, an interdisciplinary team discussed each case to determine if surgical or endovascular therapy was required based on aneurysm size, configuration, location, and the patient’s condition.

### Anesthesia

General anesthesia was induced with a bolus of propofol (2–3 mg/kg), sufentanil (30 µg), and additionally 70 µg for Mayfield clamp fixation. A medium-acting muscle relaxant (cisatracurium, 0.1 mg/kg bolus) was administered for intubation purposes only. General anesthesia was maintained with propofol (5–8 mg/kg/h) and remifentanil (0.3–0.5 µg/kg/h).

### Surgery

During surgery, after aneurysm exposure, parent and branching arteries were identified. Microvascular Doppler ultrasonography (Vascular Doppler Systems, Mizuho, Tokyo, Japan) was used before and after clip ligation (Sugita titanium aneurysm clips, Mizuho, Tokyo, Japan or Yasargil Clips, Peter Lazic GmbH, Tuttlingen, Germany). If clip positioning deemed satisfactory, ICGVA was performed, and the video analysis was then correlated with the findings from visual inspection and parent artery Doppler ultrasonography. At the same time, intraoperative monitoring including MEPs and SEPs was recorded, and in case of pathological events, the neurosurgeon was informed immediately. Intraoperative unenhanced CT, iCTA, and iCTP were performed as described elsewhere [23, 30–32].

### Indocyanine green videoangiography

All operations were performed using a microscope-integrated infrared sensitive monochrome video camera (OPMI Pentero with INFRA-RED800, Zeiss, Oberkochen, Germany). Fluorescent dye (indocyanine green, Verdy®; Diagnostic Green GmbH, Aschheim-Dornach, Germany) was administered intravenously (10 mg per dose, 0.2–0.5 mg/kg body weight) as described previously [28]. Images were continuously displayed to evaluate visualization quality and initial dye inflow. Real-time flow in arteries, branching vessels, and perforators was observed, as well as flow analyses were performed. Repeated flow analyses were performed until the clipping result was sufficient.

#### IONM

IONM was performed as previously described according to specific requests of the vascular neurosurgeon and the availability of the neuromonitoring team [33, 34]. Warning was given if three consecutive MEP showed a marked isolated increase of stimulation intensity > 20% for the respective hemisphere and amplitude decrement > 80% and/or cortical SEP amplitudes showed an amplitude decrement > 50%.

### Intraoperative computed tomography

ICT was performed using Siemens SOMATOM Definition AS + , Siemens Healthineers (Siemens AG, Munich, Germany) as described previously [11, 23, 30–32]. Patients were positioned on carbon-made radiolucent surgical tables (TruSystem 7500, Trumpf Medical), and patient’s head was fixed in a radiolucent head clamp (Mayfield radiolucent skull clamp, A-2002, Integra) [3]. Surgery was performed using continuous IONM (if available), ICGVA, and microDoppler ultrasonography. Immediately after clip placement, the table was placed in the previously saved scanning position. Subsequently, CT and automated contrast agent/saline injection were performed within 90–120 s. 3D postprocessing of iCTA/dynamic perfusion analysis of iCTP data set was achieved within 3 min by a technical assistant. During scanning, the surgical field was secured by a sterile drape. Intraoperative workflow and sterility of the operating field were not affected in any case due to unexpected collision of the patient/table and CT gantry. After data acquisition/reconstruction (in total ~ 5 min), the neuroradiologist reviewed iCT and immediately reported any pathologic findings to the neurosurgeon. Mismatch was defined as prolonged mean transit time (MTT) with moderately reduced CBF and near-normal/increased CBV. According to the CT findings, the operative field was reinspected in case of a mismatch, and the clip was repositioned when evident stenosis could be detected. In all other cases, conservative treatment, e.g., elevation of mean arterial pressure, application of anticonvulsants, nimodipine, and/or anticoagulation, was applied.

### Statistical analysis

Continuously scaled variables were analyzed with the Mann–Whitney *U* test and categorical variables with chi-square or Fisher’s exact test. Factors associated with unfavorable neurological outcome < 6 h postoperatively and at last FU were identified using univariate analysis. Significant variables were included in multivariate logistic regression analysis. A *p*-value ≤ 0.05 was considered significant. All calculations were performed using SPSS software package (Version 25).

## Results

### Patient characteristics

**Fig. 1:**
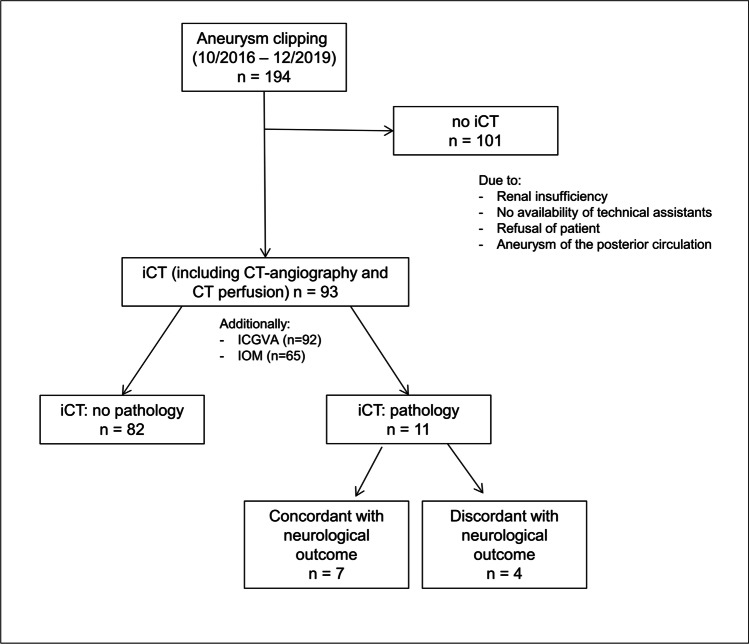
Patient inclusion

Patient inclusion is shown in Fig. 1. Study reference point was the date of clipping. Last FU was in June 2020. Patient and aneurysm characteristics are summarized in Table 1. Of 195 consecutive patients, 93 patients were studied, including 82 women and 11 men with a mean age of 58.0 years (range: 28–82 years). These patients underwent treatment for 100 aneurysms, including 96 unruptured and 4 ruptured aneurysms. Ruptured aneurysms could only be included if iCT was available during normal working hours. Aneurysms were located at MCA (48%), ICA (14.0%), ACA (11%), ACoA (19.0%), and PCoA (8.0%). Mean aneurysm size was 6.5 mm (range 2–26 mm). Temporary clipping was necessary in 13 patients (13.0%) with median temporary clipping time of 6.5 min. Median FU time was 4.4 months.

**Table 1:** Patient and aneurysm characteristics

Characteristics	All patients (*n* = 93)	No deficit (*n* = 82)	Immediate postoperative deficit (*n* = 11)	*p* value
Median age [years]	58.0	57.4	59.2	0.389
Age range [years]	28–82			
Gender (female/male)	82/11 (88.2/11.8)	73/9 (89.0/11.0)	9/2 (81.8/18.2)	0.384
SAH	4 (5.7)	3 (3.7)	1 (9.1)	0.401
Multiple aneurysms	7 (7.5)	6 (7.3)	1 (9.1)	0.599
Characteristics	All aneurysms (n = 100)	Aneurysms of pat. with no deficit (*n* = 88)	Aneurysms of pat. with immediate postoperative deficit (*n* = 12)	*p* value
Aneurysm location				0.08
MCA	48 (48.0)	44 (50.0)	4 (33.3)	
ICA	14 (14.0)	10 (11.4)	4 (33.3)	
ACA	11 (11.0)	11 (12.5)	0 (0)	
ACoA	19 (19.0)	15 (17.0)	4 (33.3)	
PCoA	8 (8.0)	8 (9.1)	0 (0)	
Side (right/left)	49/32 (60.5/39.5)	46/27 (63.0/37.0)	3/5 (37.5/62.5)	0.154
Median/mean size [mm]	6.0/ 6.5	6.0/ 5.9	6.7/ 8.5	0.003
Size range [mm]	2–26			
Aneurysm size > 5 mm	57 (57.0)	46 (52.3)	11 (91.7)	0.008
Temporary clipping	13 (13.0)	9 (10.2)	4 (33.3)	0.048
Median temporary clipping time [min]	6.5	5.1	12.3	0.024
Temporary clipping time range [min]	2–28			

*MCA* middle cerebral artery, *ICA* internal carotid artery, *ACA* anterior cerebral artery, *ACoA* anterior communicating artery, *PCoA* posterior communicating artery, *SAH* subarachnoid hemorrhage

Patients were classified if they had any immediate neurological impairment including seizures < 6 h postoperatively (*n* = 11) or not (*n* = 82). Patients with occurrence of a neurological deficit differed from patients without deficit in aneurysm location at ICA (33.3% vs. 11.4%, *p* < 0.08) and AComA (33.3% vs. 17.0%, *p* < 0.09), more aneurysms with size > 5 mm (91.7% vs. 52.3%, *p* < 0.008), more frequent temporary clipping (33.3% vs. 10.2%, *p* < 0.048), and longer temporary clipping time (12.3 vs. 5.1 min, *p* < 0.024).

#### ICGVA

**Fig. 2:**
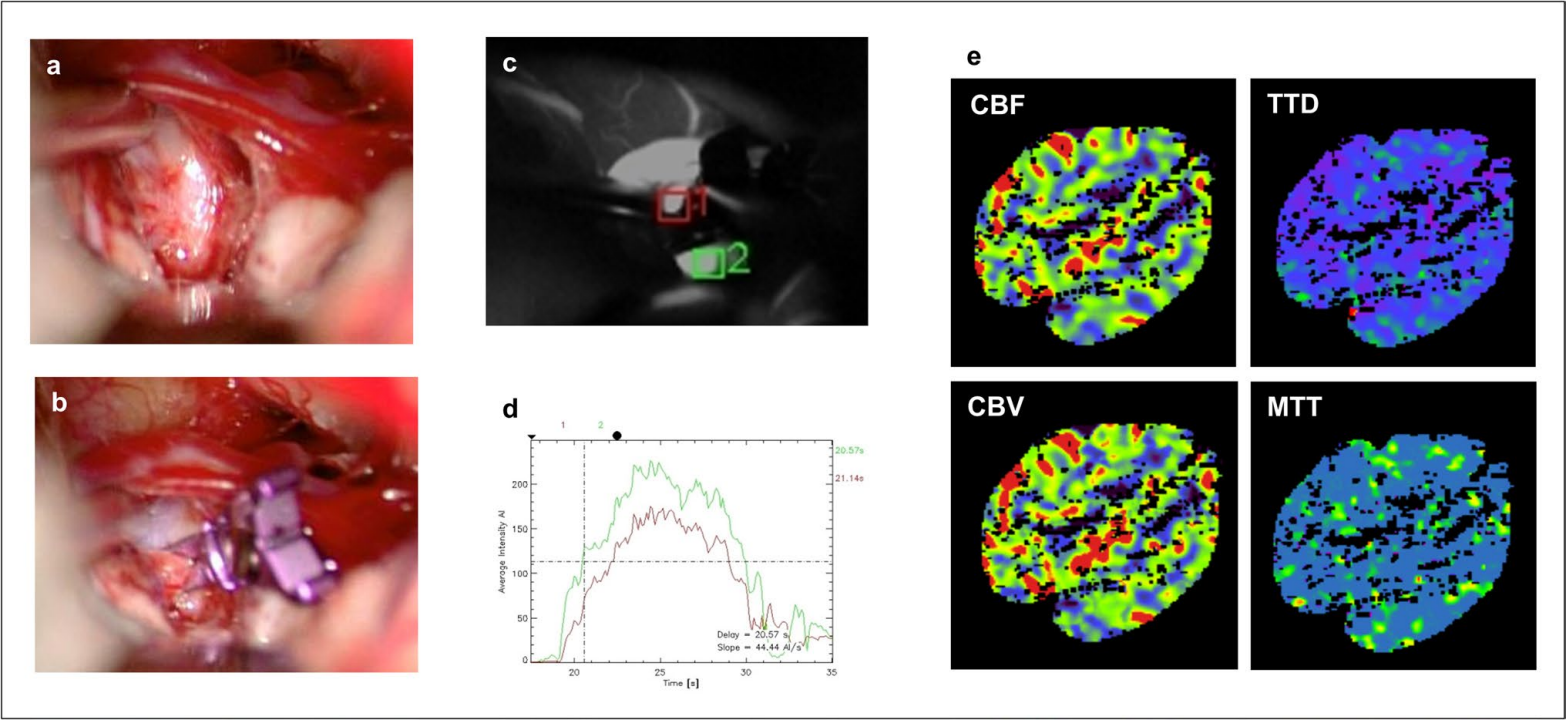
A 70-year-old female patient with an incidental irregularly configurated ACoA aneurysm was operated (**a** before clipping, **b** after clipping). Intraoperative ICGVA showed a difference in flow velocity of the two A2 branches (**c**, **d**). However, iCTP showed no difference in CBF, CBV, TTD, or MTT (**e**)

ICGVA was applied in 92/93 patients (98.9%) without adverse events showing regular enhancement of the arterial anatomy in close vicinity to the clip. In one patient suffering from glucose dehydrogenase deficiency, ICGVA was not applied. ICGVA flow analysis showed regular contrast enhancement of the parent vessel and the distal branches in all 92 cases. However, there were differences in the flow velocity of the distal branches in several cases, although intraoperative CTP could not confirm a mismatch in MTT. An exemplary case is shown in Fig. 2. A 70-year-old female patient with an incidental irregularly configurated ACoA aneurysm was operated on. Intraoperative ICGVA showed a difference in flow velocity of the two A2 branches. Reinspection revealed no clip stenosis. Also, iCTP showed no difference in CBF, CBV, TTD, or MTT.

### IONM changes

In 66/93 patients (60 females/6 males) with 71 aneurysms, IONM was performed. Aneurysms were located at MCA in 38 (57.6%), ICA in 7 (10.6%), ACA in 8 (12.1%), ACoA in 14 (21.2%), and PCoA in 4 patients (6.0%). In all patients, SEP/MEP were recorded for the relevant vascular territory. In all but two patients (3.0%), IONM was unchanged compared to the pre-clipping measures.

In detail, in one patient (Table 2, no. 4) contralateral MEP loss and significant transient decrement of median nerve SEP amplitudes occurred immediately after first permanent clip placement. Meticulous inspection of the surgical site led to identifying an impaired perforating artery in the vicinity of the aneurysm. Unfortunately, repositioning of the clip did not lead to recovery of signals and the patient suffered from hemiparesis.

**Table 2:** Patients with immediate postoperative neurological deficit and the corresponding results of intraoperative CTA, CTP, ICGVA, and IOM

No	Age [years]	Sex	SAH	Location	Size [mm]	iCTA	iCTP	ICGVA	IOM	Resulting ischemia postop (CT/MRI)	Outcome postop (< 6 h)	mRS postop	Outcome at discharge	mRS one week	Outcome 8 weeks	mRS 8 weeks
Normal iCTP, but early neurological deficit																
1	69	F	N	ACoA	6.7	Normal	Normal	Normal	Normal	Fornix ischemia	Disoriented	2	Memory deficit	2	Memory deficit	1
2	52	F	N	ACoA	8	Normal	Normal	Normal	Normal	Fornix ischemia	Disoriented	3	Disoriented	3	Memory deficit	2
3	50	F	N	ICA	6.7	Normal	Normal	Normal	Missing	Ischemia left capsula interna	Severe hemiparesis	4	Reduced fine motor skills	1	Reduced fine motor skills	1
4	50	M	N	ICA	6.3	Normal	Normal	Normal	Impaired	Mismatch in postoperative iCTP and ischemia left capsula interna	Severe hemiparesis	4	Severe hemiparesis	4	Slight hemiparesis	2
Mismatch in iCTP harboring conservative treatment strategies																
5	59	F	N	MCA	6	Normal	Slight mismatch (temporal)	Normal	Normal	No ischemia	Slight hemiparesis and aphasia	3	No deficit	0	No deficit	0
6	68	F	N	ACoA	10	Normal	Mismatch (prolonged TTD frontal)	Normal	Missing	Delayed venous congestion with bleeding	Memory deficit	2	Memory/cognitive deficit	2	Memory deficit	2
7	66	F	N	ICA	6.7	Impaired	Mismatch (frontal)	Normal	Missing	Cortical diffusion restriction right frontal lobe	Seizures	1	No deficit	0	No deficit	0
8	55	F	N	MCA	6	Impaired	Mismatch (frontal)	Normal	Normal	Ischemia right corona radiata	Severe hemiparesis	4	Slight hemiparesis	2	Slight hemiparesis	2
9	53	F	N	ICA	10	Impaired	Mismatch (prolonged MTT + TTD temporal)	Normal	Normal	ACI occlusion, but good collateralization	Slight hemiparesis	3	No deficit	0	No deficit	0
Mismatch in iCTP leading to clip repositioning and a second iCTP with improved perfusion																
10	61	M	N	MCA	26	Impaired	Mismatch (frontal)	Normal	Normal	Ischemia frontal operculum	Aphasia	3	Aphasia improved	2	Minimal speech impairment	1
						Improved	Normal									
11	71	F	Y	ACoA	7	Impaired	Mismatch (frontal)	Normal	Impaired	No ischemia in both Acom territories	GCS3	5	GCS3	5	GCS8	4
						Improved	Normal									

In the other patient (Table 2, no. 11), slow deterioration was observed of left abd. hallucis MEP with amplitude decrement and increment in stimulation intensity with some delay to the permanent clip application and during iCT imaging. Therefore, clip repositioning was performed and MEP improved. The postoperative course required long-term intensive care treatment due to excessive SAH-associated vasospasm and multiple cerebral infarctions.

### Intraoperative CT findings

Detailed patient, imaging, and surgery-related data and neurological outcome are illustrated in Table 2. In all 93 patients, image quality was technically adequate for evaluation. In detail, unenhanced brain CT showed no signs of infarction/acute bleeding in all cases. In 86/93 patients (92.5%), both iCTA and iCTP did not reveal any pathological findings. In cases of temporary clipping, there was no mismatch in iCTP detectable. Mean time for iCT performance and analysis was 5–8 min and radiation protocol comprehended in total 3.67mSV.

In 5 patients (Table 2, nos. 7–11), iCTA showed impaired structural anatomy of dependent vessels, while iCTP showed pathological perfusion. In 2 patients, iCTA was unremarkable, while iCTP showed an impending perfusion deficit (nos. 5, 6). In all patients with pathologic findings on iCTA/iCTP, the surgical site was inspected immediately to rule out relevant clip stenosis. In none of these patients, an unanticipated complete vessel occlusion was found which was in accordance with normal ICGVA findings.

In 4 patients (nos. 1–4) who developed neurological deficits postoperatively, iCTP showed normal CBP. In detail, 2 patients (nos. 1, 2) developed memory deficits consistent with a fornix ischemia detected in postoperative CT. Despite unremarkable iCT, two patients (nos. 3, 4) suffered from initial severe hemiparesis which gradually improved during FU due to an ischemia in the area of the ipsilateral internal capsule detected in postoperative CT.

In 5 patients (nos. 5–9), intensified conservative treatment as aggressive as justifiable was immediately initiated. One patient (no. 5) with slight iCTP mismatch and normal iCTA suffered from transient aphasia and hemiparesis which resolved completely until discharge. The venous flow analysis in iCTP was pathologic in one patient (increased TTD). In this patient, no immediate reaction was judged necessary by the responsible neurosurgeon. However, even though CT on day 1 post-OP was unremarkable, CT on day 3 showed an intraparenchymatous hemorrhage caused by venous congestion necessitating surgery (no. 6). In two patients (nos. 7, 8) with pathological iCTP findings, clip position and dependent vessel anatomy were found regular in inspection and iCTA; thus, a thromboembolic event was suspected. The postoperative imaging revealed an ischemia in the corresponding area of the intraoperatively detected mismatch in iCTP. In one patient (no. 9), it was not feasible to clip a fusiform, twisted aneurysm neck at the ICA siphon without occlusion of ICA. This was tolerated due to excellent collateralization confirmed by iCTA and unremarkable IONM.

**Fig. 3:**
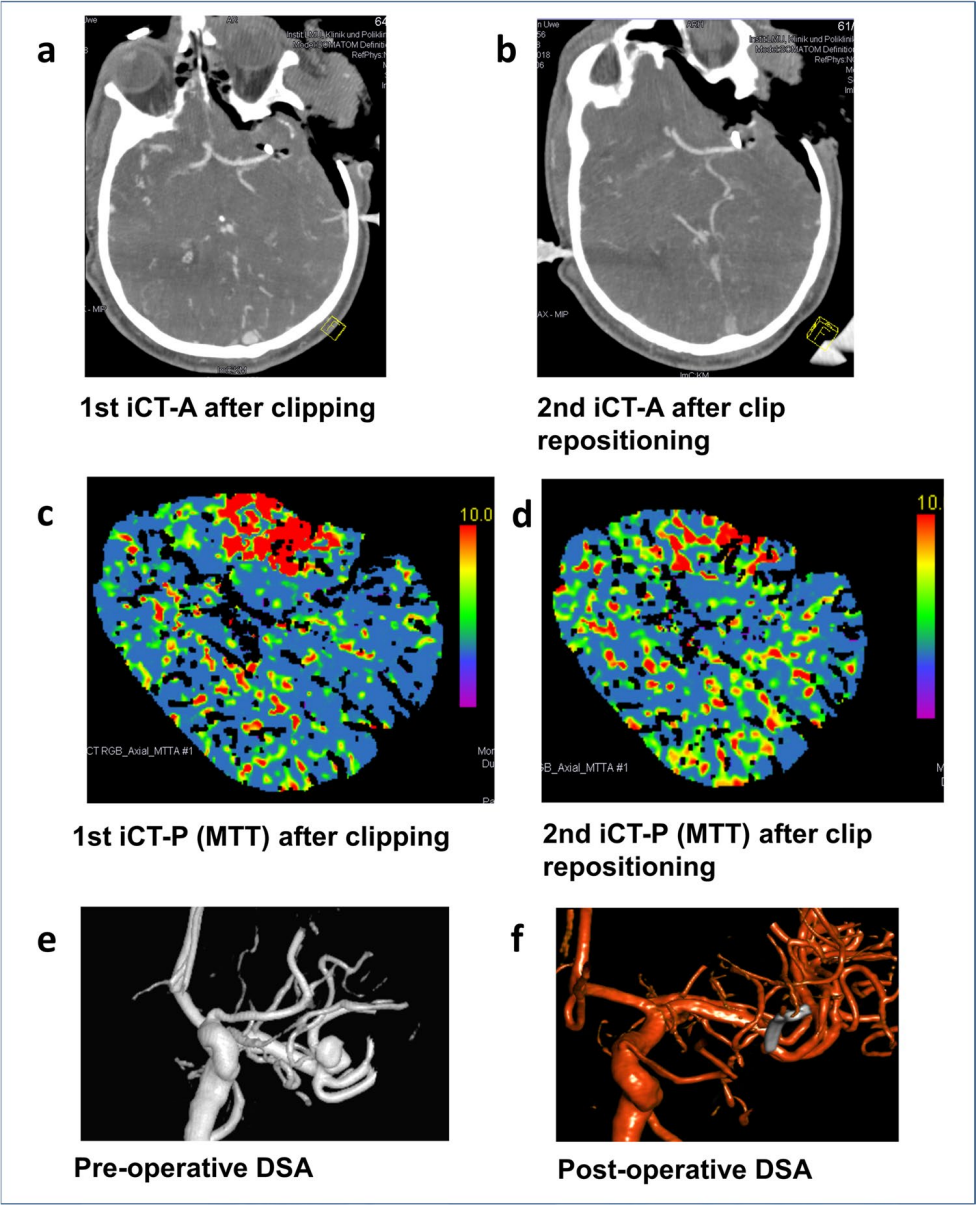
A 61-year-old male patient presented with a partially thrombosed and calcified left-sided giant MCA aneurysm (**e**). After clipping, iCTA showed a reduced contrast enhancement in one M2 branch in close vicinity of the clip (**a**). iCTP showed a mismatch by means of an increased MTT in the frontal M2 vessel territory (**c**). Therefore, the clip was repositioned, and a second iCTA showed normal vessel enhancement (**b**), and a second iCTP showed normal MTT compared to the contralateral side (**d**). Postoperative angiography showed a complete occlusion of the aneurysm (**f**). The patient presented with a transient aphasia during the early postoperative course that almost completely resolved until last FU

In two patients, iCTP mismatch led the neurosurgeon to thorough reinspection of the surgical field resulting in clip repositioning necessary (nos. 10, 11). In detail, one patient suffers from a large MCA aneurysm; the aneurysm sack had to be excised due to torquing of the parent vessel resulting in increased MTT in iCTP (no. 10). The second iCTP after removal of the aneurysm sack showed normalization of MTT (Fig. 3). One patient (no. 11) with a ruptured ACoA aneurysm showed reduced CBP in iCTP, which resulted in clip repositioning and a subsequently improved iCTP. However, this patient did not improve neurologically due to severe vasospasm resulting into infarction.

In summary, iCTP mismatch resulted in an intensified conservative treatment in five patients (5.3%) and in clip repositioning in two patients (2.2%). In these patients, neurological deficits improved until last FU. Four patients (4.3%) suffered from neurological deficits, even though iCTP was normal. This was attributed to clip artifacts and undetectable small ischemic lesions in the internal capsule.

### Neurological outcome

**Fig. 4:**
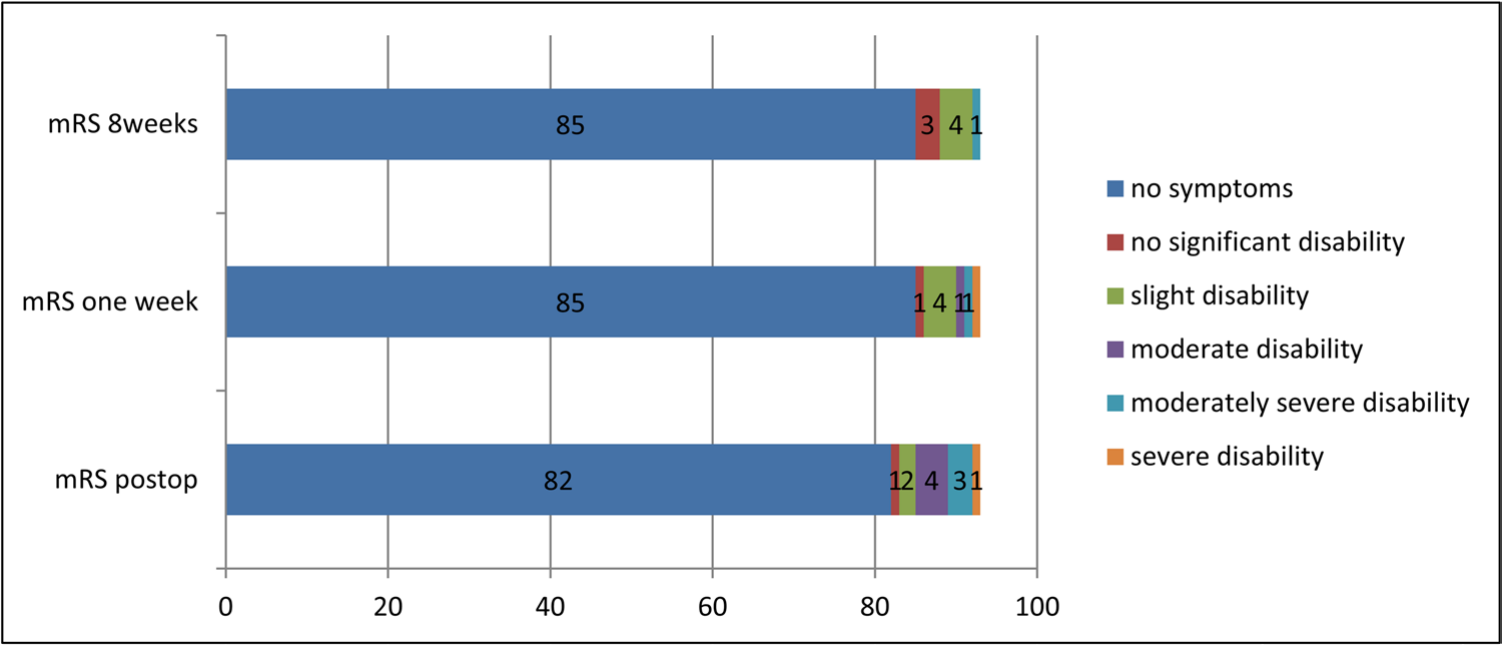
Outcome scales according to the modified Rankin scale (mRS) immediately postoperatively (within 6 h after operation), at discharge and at last FU

Immediately after surgery, 11 patients (11.8%) presented a transient clinical deterioration with mRS0-3 *n* = 7 (7.5%) and mRS4-5 *n* = 4 (4.3%). At discharge, neurological symptoms had completely resolved in 4 patients (4.3%), improved in 4 patients (4.3%), and remained stable in 3 patients (3.2%). At last FU, 3 patients (3.2%) had recovered completely with mRS0 (in total: 91.4%), 7 patients (7.5%) presented with mRS1-2, whereas one patient (1.1%) with SAH who was admitted with GCS3 remained with mRS4 (Fig. 4).

### Independent predictors for neurological deterioration

Relevant patient- or surgery-related variables were evaluated stepwise in bivariate and multivariate analyses (Table 3). Accordingly age (*p* < 0.03), side (*p* < 0.05), aneurysm size (*p* < 0.02), and iCTP mismatch (*p* < 0.001) were independent risk factors for neurological deterioration < 6 h postoperatively. In the multivariate analysis, iCTP mismatch remained statistically significant (*p* < 0.001). Risk factors for impaired neurological outcome at last FU were iCTP mismatch (*p* < 0.04) and use of temporary clipping (*p* < 0.002). In the multivariate analysis, temporary clipping remained statistically significant (*p* < 0.002).

**Table 3:** Uni- and multivariate analysis for postoperative outcome and outcome at last follow-up

	Outcome postoperatively		Outcome at last FU	
	*p* value	OR/95% CI	*p* value	OR/95% CI
Univariate analysis				
Age (continuously)	< 0.03	1.070/1.008 to 1.136	NS (*p* = 0.72)	
Age (< 50 vs. ≥ 50 years)	NS (*p* = 0.58)		NS (*p* = 0.20)	
Gender (male vs. female)	NS (*p* = 0.18)		NS (*p* = 0.26)	
Side (right vs. left)	< 0.05	0.183/0.034 to 0.979	NS (*p* = 0.17)	
Aneurysm size (< 5 mm vs. ≥ 5 mm)	NS (*p* = 0.99)		NS (*p* = 0.15)	
Aneurysm size (continuously)	< 0.019	1.514/1.071 to 2.139	NS (*p* = 0.66)	
Aneurysm location	NS (*p* = 0.57)		NS (*p* = 0.42)	
iCTP mismatch	< 0.001	0.030/0.006 to 0.144	< 0.035	0.178/0.036 to 0.886
IOM	NA*		NA*	
Temporary clipping (yes/no)	NS (*p* = 0.07)		< 0.002	0.078/0.015 to 0.392
Temporary clipping time	NS (*p* = 0.10)		NS (*p* = 0.43)	
Multiple aneurysms	NS (*p* = 0.94)		NS (*p* = 0.08)	
Multivariate analysis				
iCTP mismatch	< 0.001	0.012/0.001 to 0.128		
Temporary clipping			< 0.002	0.078/0.015 to 0.392

^*^No significant results according to low number of cases

*FU* follow-up, *iCTP* intraoperative CT perfusion, *IOM* intraoperative monitoring, *NS* not statistically significant, *NA* not available, *OR* odds ratio, *95% CI* 95% confidence interval

### Sensitivity/specificity

The specificity, sensitivity, and accuracy for diagnosing an immediate postoperative deficit by iCTP were 100%, 63.6%, and 95.7% and by iCTA were 100%, 45.5%, and 93.5%, respectively (Table 4). With IONM as a reference, specificity, sensitivity, and accuracy for diagnosis of an immediate postoperative deficit were 83.3%, 18.2%, and 86.4% (Table 4). The estimated number needed to treat to prevent postoperative neurological deficits by iCTP was 13, and the absolute risk reduction using iCTP was 7.5%.

**Table 4:** Sensitivity, specificity, PPV, NPV, FN, FP, and accuracy for the detection of an immediate postoperative neurological deficit by iCTP, iCTA, and IONM

Parameter	Sens [%]	Spec [%]	PPV [%]	NPV [%]	FN [%]	FP [%]	Accuracy [%]
iCTP	63.6	100	100	95.3	36.4	0	95.7
iCTA	45.5	100	100	93.2	54.5	0	93.5
IONM	18.2	83.3	100	85.9	81.8	0	86.4

*iCTP* intraoperative CT perfusion, *iCTA* intraoperative CT angiography, *IONM* intraoperative monitoring, *Sens* sensitivity, *Spec* specificity, *PPV* positive predictive value, *NPV* negative predictive value, *FN* false negative, *FP* false positive

### Complications and side effects

One patient (1.1%) needed re-surgery due to venous congestion and subsequent intracerebral hemorrhage (see above). Two patients (2.2%) with postoperative CSF fistula were successfully treated by spinal drainage/pressure bandage. Two patients (2.2%) had wound healing disorders which resolved under antibiotic treatment. Two patients (2.2%) suffered from pulmonary embolism needing anticoagulation. One patient (1.1%) developed a mild contrast agent allergy during iCTA/iCTP not requiring further treatment.

## Discussion

In modern interdisciplinary neurovascular centers, aneurysm clipping is a well-established procedure with low morbidity and excellent long-term results. To further reduce procedure-related complications, numerous technical improvements have been introduced, like ICGVA or IONM [1, 6, 9, 28, 29]. Nevertheless, easily overlooked affection of distant vessels by local vasospasm, detachment of microcalcifications, or relative clip stenoses pose a relevant but possibly avoidable risk for neurological deterioration [21]. The aim of the study was to investigate whether iCT, iCTA, and iCTP can provide relevant data on brain perfusion in a timely manner, so that appropriate conservative or surgical countermeasures can be taken in the operating room to improve patient outcome.

In previous studies, we were able to demonstrate the feasibility of iCT and its integration into the intraoperative workflow in a double-room installation in selected cases [10, 30–32]. Here, we show for the first time the seamless integration of the iCT in a large neurovascular patient cohort. Mean time for iCT performance and analysis was 5–8 min and radiation protocol comprehended in total 3.67mSV—comparable to a 4-vessel catheter angiogram. Also, adverse events were rare—only one patient developed a circulative, but not allergic reaction to contrast agent resolving under short-term catecholamine therapy.

Patient and aneurysm-specific characteristics were in line with other studies with predominance of female patients, MCA aneurysms, and median aneurysm sizes of 6–7 mm [15, 16]. However, a potential selection bias was caused by the restricted availability of radiological technical assistants exclusively during normal working hours and by exclusion of posterior circulation aneurysms [5].

In other studies, treatment-related ischemia after aneurysm clipping occurred in 9–20% [2, 12, 14, 16, 17, 22]. In a large study of patients with unruptured intracranial aneurysms (UIA), surgery-associated complication rate was 18% with only 2.1% permanent neurological deficits after clipping [12]. In a former trial from the ISUIA investigators, intracranial hemorrhage was reported in 4% and cerebral infarction in 11% of patients [36]—total morbidity/mortality rates at 1 year were 15.7% [14]. However periprocedural ischemia represented a significant risk factor with strong clinical impact on outcome, which had been underestimated in the literature so far [21]. In our study, 11 patients (15.7%) showed neurological impairments immediately after surgery. At last FU, only one patient with SAH who was admitted with GCS3 remained with mRS4 (1.1%), 7.5% presented with mRS1-2, and all other patients with a postoperative deficit resolved completely. In accordance to other studies, patient characteristics/factors associated with unfavorable outcome were aneurysm size/location, presence of SAH, and temporary clipping time [24, 26].

In our study cohort, benefits of iCT were especially observed in cases where the microscopic field was very narrow, e.g., in Acom aneurysms where ICGVA suggested the suspicion of discrete flow velocity differences of the distal branches, but no proof of definite vessel occlusion. In two cases, ICGVA was unable to detect relative clip stenosis, whereas iCTP mismatch in the corresponding distinct vascular territory led to effective intraoperative clip repositioning and CPB improvement, confirmed in a second iCTP. We assume that clinically relevant infarction could be prevented in these patients, who achieved favorable neurological outcome. In the other five cases with a reduced CBP in iCTP, inspection of the surgical field and the clip position did not show useful options for clip repositioning. An aggressive conservative therapy was thereupon initiated at the earliest possible time where the surgical team assumed best effectiveness on salvageable tissue at risk. None of these patients showed an impaired neurological outcome. Further relevant information provided by iCT were the detection of a sufficient collateralization in a case where ICA occlusion deemed intraoperatively necessary. Also, in another case, the detection of a venous congestion demonstrated in a prolonged venous time to drainage (TTD) was predictive for secondary intraparenchymatous hemorrhage.

Limitations of iCT were observed especially in the detection of small ischemic lesions caused by perforating vessel occlusions and in close vicinity to clip artifacts. In this study, we missed to anticipate clinically relevant ischemia in 4 patients with postoperative demarcated infarctions in the internal capsule and fornix (2 patients each). Circumscribed regions of interest (ROI) and insufficient resolution for very small perforators could contribute to this limitation. This is in accordance to former studies in stroke patients, where CTP had high specificity in detection of lacunar strokes, though its sensitivity was low, especially for localization in basal ganglia and thalamus [4, 18–20, 25]. Therefore, technical developments should focus on improved resolution techniques and programs for clip artifact suppression especially in eloquent areas to save potentially salvageable tissue.

In contrast to ICGVA and iDSA, which directly show anatomical preservation of vessel patency, iCTP illustrates dynamic flow-associated CBP. Even in this cohort with few cases of treatment-associated ischemia, there was evidence for beneficial information based on iCT compared to ICGVA, where ICGVA would have missed a relevant clip stenosis in two patients. Certainly, iDSA points out local anatomy of the clip construct and anatomical preservation of dependent vessels in highest quality, but this method is an invasive procedure which requires special logistic effort in specialized centers [1, 6, 9]. Besides intraoperative imaging tools, IONM is useful to monitor motor pathway integrity and to predict postoperative motor status [7]. However, the assumed positive impact of introducing SSEP/MEP monitoring on overall neurological outcome in elective microsurgical clipping of unruptured intracranial aneurysms did not reach significance as recently shown [13]. False-negative and false-positive findings in MEP were generated by deep white matter stimulation potentially bypassing ischemic lesion or by CSF drainage and pneumatocephalus [7]. In our setting, tailored and slight suprathreshold stimulation was applied to minimize the risk of activating the motor pathway below the level of the internal capsule. Nevertheless, IONM detected permanent postoperative deficit with low sensitivity and specificity compared to iCT which points in the same direction as data from other groups [27]. A recent study showed that postoperative ischemia caused by clip stenosis could be reduced by ultrasound-based transit time flowmetry. A flow < 40% of the baseline measurement significantly predicted clip-related ischemia and a flow less than 50% from baseline was an independent predictor of unfavorable outcome [35]. Therefore, it would be interesting for the future to compare results of the iCT with transit time flowmetry if there is beneficial information of iCT especially in the detection of small ischemic lesions caused by perforating vessel occlusion.

We conclude that iCT provides the neurosurgeon with relevant information about vessel’s patency and CBP and therefore enables to promptly optimize intraoperative strategy and adapt the therapeutic management. Especially in cases with complex partially thrombosed or calcified aneurysms or a narrow microscopical field, we assume that iCTP can provide beneficial information for detecting relevant mismatches in the affected distant vascular territory.

## Conclusion

In summary, we show for the first time in a large patient cohort that iCT can be integrated into daily routine in clipping procedures without disturbance of operative workflow and without occurrence of severe adverse events. Moreover, we were able to demonstrate that iCTP is reliable to detect critical CBP and enables the neurosurgeon to react by clip repositioning or immediate application of aggressive conservative treatment. ICT is a valuable addition to the armamentarium of a multimodal intraoperative diagnostics including ICGVA and IONM in aneurysm surgery to minimize risks of treatment-related ischemia and improve patient outcome.

## Abbreviations

CBP: Cerebral blood perfusion
CBV: Cerebral blood volume
CSF: Cerebrospinal fluid
CT: Computed tomography
DSA: Digital subtraction angiography
ICG-VAG: Indocyanine green videoangiography
iCT: Intraoperative CT
iCTA: Intraoperative CT angiography
iCTP: Intraoperative CT perfusion
iDSA: Intraoperative DSA
FU: Follow-up
IONM: Intraoperative neuromonitoring
MEP: Motor evoked potential
MRI: Magnetic resonance imaging
mRS: Modified Rankin score
MTT: Mean transit time
SEP: Somatosensory evoked potential
SAH: Subarachnoid hemorrhage
TTD: Time to drain
